# Thoracic Outlet Syndrome: Biomechanical and Exercise Considerations

**DOI:** 10.3390/healthcare6020068

**Published:** 2018-06-19

**Authors:** Nicholas A. Levine, Brandon R. Rigby

**Affiliations:** Biomechanics and Motor Behavior Laboratory, Department of Kinesiology, Texas Woman’s University, Denton, TX 76207, USA; nlevine@twu.edu

**Keywords:** functional anatomy, hypermobility, joint, ligament, mechanics, muscle, shoulder

## Abstract

Thoracic outlet syndrome (TOS) describes a group of disorders that are due to a dynamic compression of blood vessels or nerves, between the clavicle and first rib or cervical vertebral nerve roots. Individuals with TOS typically experience upper limb pain, numbness, tingling, or weakness that is exacerbated by shoulder or neck movement. The causes of TOS vary, and can include abrupt movements, hypertrophy of the neck musculature, and anatomical variations in which the brachial plexus roots pass through this musculature, edema, pregnancy, repeated overhead motions, the blockage of an artery or vein, or abnormal posture. To understand the complexity of this condition, an analysis of shoulder anatomy and mechanics are needed to help describe limitations and the subsequent pathophysiology of TOS. Several treatment options are available, including surgery, medications, and exercise. A comprehensive study of shoulder anatomy and biomechanics, and knowledge of the benefits of exercise, may help clinicians and healthcare practitioners determine the most appropriate treatment plan for an individual with TOS.

## 1. Introduction

Thoracic outlet syndrome (TOS) is defined as the compression of nerves or blood vessels near the base of the neck [1]. Specifically, the compression of the brachial neural plexus, subclavian artery, subclavian vein, or any combination of these vessels, may occur [2,3,4,5,6,7,8]. Compression of the subclavian artery or vein is classified as vascular TOS (vTOS) [9]. The condition is caused by the presence of a cervical rib or bony growth near a nerve root in the neck [2,9,10,11,12,13]. This abnormality may alter the attachment site of the scalenes, which may further complicate vTOS [1,5,6,10,14,15]. Compression or irritation of brachial nerves from a cervical rib is referred to as true neurological TOS (nTOS) [2,9,16]. Other forms of TOS, which are often grouped together and referred to as non-symptomatic TOS, are identified when there are no apparent abnormalities from standard imaging techniques (e.g., X-ray, MRI), manual manipulation assessments (e.g., upper limb tension test of Elvey, Adson’s test), or neurological conduction tests (e.g., electroneuromyography) [4,7,9]. Those with non-symptomatic TOS typically share the same symptoms as neurological TOS [17]. The difficulty in diagnosing nTOS can be due to anatomical variations (e.g., the brachial plexus nerves piercing the scalenes) [18]. This causes a predisposition to nTOS, and is commonly not found in standard nTOS examinations [18].

Symptoms of TOS may include muscle weakness, paresthesia, discoloration, swelling, numbness, and pain in the hand or arm, and muscle atrophy (particularly in the hand) (e.g., [2,8,9,14]). There is generally no agreement on the etiology of TOS, which can make prescribing treatment for the condition difficult. Proper physical examination of the patient can help differentiate between the various forms of TOS, and other conditions that elicit similar symptoms of TOS (i.e., carpal tunnel syndrome or pectoralis minor syndrome) [17,19,20]. Approximately 90% of all TOS cases are diagnosed as nTOS [4,11,12]. The majority of vTOS cases are diagnosed as arterial vTOS [4]. Thoracic outlet syndrome is typically diagnosed in early adulthood (i.e., ages 20–40 years), and is more prevalent in those with the shoulder flexed for majority of the work day, have repeated trauma to the shoulder joint, and with those who exhibit abnormal posture, including positions required to play bowed instruments [11,15]. Repeated trauma to the head or neck, postural dysfunction, extended duration in compromising shoulder positions, pregnancy, edema, anatomical deviations, hypertrophied muscles (e.g., scalenes), boney growths, and muscle weakness are all theorized to be contributing factors to TOS [2,4,9,11,12,13,18]. The most commonly presented cause of TOS appears to be an abrupt flexion-extension motion (e.g., whiplash) at the atlantoaxial joint and other joints between the cervical vertebrae, which can lead to symptoms present with both vTOS and nTOS [2,9,16,21]. This whiplash motion can result in instability at the atlantoaxial joint, causing the surrounding musculature to shorten, in order to compensate for the laxity in the joint. Specifically, the sternocleidomastoid and scalenes shorten, which can alter the function of these muscles [22]. This can lead to an entrapment of the brachial plexus, subclavian artery, subclavian vein, or a combination of these vessels and tissues [2,19]. The subclavian artery and vein run posterior and inferior to the clavicle, and deep to the pectoralis minor [19]. Both the clavicle and pectoralis minor muscle can influence shoulder motion. Therefore, it is important to understand the functional anatomy of the shoulder, how the shoulder affects TOS, and how rehabilitation efforts focused on shoulder and back musculature can provide a conservative treatment plan.

## 2. Functional Anatomy of the Shoulder

### 2.1. Shoulder Components

The shoulder joint is complex, composed of four distinct joints [23]. The primary shoulder joint actions include flexion/extension, abduction/adduction, and internal/external rotation, allowing for a total of three rotational degrees of freedom (DOF). The shoulder is typically characterized as having a total of six DOF, three rotational and three translational [24]. The glenohumeral (GH) joint is the connection of the humeral head and the glenoid fossa, and is responsible for most of the flexion/extension motion allowed [25]. The GH joint also allows for abduction/adduction and internal/external rotation [26]. Since the glenoid fossa and head of the humerus are only partially connected at the GH joint, the shoulder exhibits a great amount of mobility, while sacrificing stability [23,24]. The sternoclavicular (SC) joint is a saddle joint, and serves as the only connection from the shoulder to the axial skeletal system [27]. The SC joint allows for several movements associated with the clavicle, including elevation/depression around the anterior-posterior axis, protraction/retraction around the vertical axis, and anterior/posterior rolling rotation around the medial-lateral axis [27]. The acromioclavicular (AC) joint is a plane joint which generally restricts movement at the shoulder and allows forces to be transmitted from the upper extremity to the clavicle [27]. The AC joint is responsible for anterior/posterior tilting of the scapula around the medial-lateral axis, upward/downward rotation around the anterior-posterior axis, and internal/external rotation around the vertical axis [26]. The scapulothoracic (ST) joint aids in the internal rotation of the shoulder [27,28,29]. It is not classified as a fibrous, cartilaginous, or synovial joint. Motion that occurs at this joint is due to the motion of the SC joint, AC joint, or a combination of the two [26]. Finally, the glenoid labrum can act as an attachment point, facilitate the generation of a negative intra-articular pressure to maintain stability, and increase the connection area with the humeral head [23,24,26].

### 2.2. Shoulder Ligaments

There are four major ligaments that act on the shoulder. The superior GH ligament originates in the supraglenoid tubercle and inserts on the lesser tubercle. The primary purpose of this ligament is to resist inferior rotation while the shoulder is adducting, and to limit the external rotation of the shoulder [23,28,30]. The middle GH ligament originates in the supraglenoid tubercle and inserts into the lesser tuberosity. The primary role of this ligament is to oppose anterior translations of the shoulder, particularity in the abducted and externally rotated positions [23,28,30]. The inferior GH ligament inserts onto the humeral neck in either a “V-like” or “C-like” shape [23]. The main purpose this ligament is to resist anterior translations and inferior translations when the shoulder is abducted [23,28]. The inferior GH ligament can be divided into three sections and is considered as one of the most important ligaments of the shoulder joint [23]. The anterior band originates from the anterior labrum, glenoid fossa, or glenoid neck [23,28]. The posterior band is involved with static stabilization and resists posterior translations of the shoulder; however, it is not found in some people [28]. The final component of the inferior GH ligament is the axillary pouch [23,28]. The axillary pouch and anterior band both prevent anterior translation of the shoulder [31]. The coracohumeral ligament originates from the base of the coracoid process, and inserts into the greater tuberosity [30]. Primary functions of the coracohumeral ligament include resisting posterior and inferior translations of the humeral head [28,30].

### 2.3. Shoulder Musculature

Arguably, the most critical muscles that influence shoulder motion and stability are the rotator cuff muscles. This muscle group is responsible for maintaining the humeral head in the appropriate position and providing the necessary torque produced from agonist and antagonist coactivations [28,32]. The muscles in this group include the supraspinatus, infraspinatus, teres minor, and subscapularis. The supraspinatus originates in the supraspinous fossa and inserts into the greater tubercle [33]. It is responsible for a large amount of abduction, up to approximately 90° [33,34]. The infraspinatus originates in the infraspinatus fossa and inserts into the greater tubercle [33]. The superior portion of the infraspinatus is typically utilized as a weak abductor, while the inferior portion acts as a stabilizer [34]. However, the infraspinatus plays a larger role as an external rotator of the shoulder [23,28,34]. The teres minor originates in the lateral border of the scapula, and inserts into the greater tubercle [33]. The teres minor complements the function of the infraspinatus [28,32,34]. The subscapularis originates in the subscapular fossa and inserts into the lesser tubercle of the humerus [33]. The subscapularis internally rotates the shoulder, and also aids in abduction [32,34]. The medial and inferior portions of the subscapularis may be critical for the stabilization of the shoulder during abduction [34]. The origin, insertion, and function other significant shoulder musculature are summarized in Table 1.

### 2.4. Other Musculature Considerations

TOS symptoms may persist due to abnormal muscle mechanics at the pelvis [36]. Pelvic alignment has been shown to affect posture, gait, and alignment of the axial skeleton (particularly the head and neck) [37,38]. Abnormal pelvic mechanics may, therefore, decrease the ability to properly perform activities of daily living and reduce quality of life. Additionally, if the axial skeleton is not aligned properly, stresses exerted on the body from exercise, occupational demands, or even from daily activities, may be distributed to other body segments and tissues that are not accustomed to adapting to these loads. For example, if the hip flexors (particularly the iliopsoas) are tight and the hip extensors are weak (particularly the glutes and hamstrings), the individual may exhibit an anterior pelvic tilt [39]. In addition, abdominal muscles (e.g., transverse abdominus) which normally generate a torque couple to resist the hip flexors, may be weak, thus causing lumbar vertebrae lordosis [39]. This lordosis may induce thoracic vertebrae kyphosis, a common condition in those diagnosed with TOS [36].

## 3. Static and Dynamic Stabilization of the Shoulder

The motion of the shoulder can be separated into static (passive) and dynamic (active) components [27,28]. Static components include the congruency between the humeral head and glenoid fossa, the fibrocartilaginous labrum, constrained capsule, GH ligaments, and the negative intra-articular pressure present in the labrum [27,28]. The glenoid fossa is a concave structure that connects to the humeral head. This connection allows for a ball-and-socket appearance. However, there is little contact between the humeral head and glenoid fossa [27]. The labrum increases this connection up to approximately 50% between the glenoid fossa and the humeral head [28]. Unique to the shoulder, the ligament and tendons of the surrounding musculature merge into one, creating a thick fibrous connection (i.e., constrained capsule) around the joint, with only the superior ligament being distinguishable from the rest of the ligaments [26]. Electromyographic activity is typically not present in the rotator cuff muscles or deltoids at rest [26], thus allowing for intra-articular pressure to create a suction cup effect between the glenoid fossa and humeral head, maintaining shoulder stability [24].

Dynamic stability is a term associated with the stabilization of the shoulder while moving continuously throughout its range of motion. It is influenced by the musculature, ligaments, and tendons present at the joint [24,28,30]. The primary muscles involved with dynamic stabilization of the shoulder are the rotator cuff and deltoid muscles, and each have unique roles depending on the shoulder motion. For example, when the shoulder abducts, the supraspinatus and medial deltoid are primarily responsible for the motion; however, when the shoulder is elevated, the infraspinatus, subscapularis and teres minor are responsible for maintaining the humeral head in position [26]. To allow for full abduction, the humeral head undergoes four times greater movement (rotation and slight translations) when compared to the scapula within the first 30° of motion. This relationship is reduced to a 2:1 humeral head-to-scapula ratio for movements greater than 30° of abduction [26]. The SC and AC joints also move to allow for full abduction of the shoulder [26]. The combination of the movements of these shoulder components illustrate the concept of dynamic stabilization, which allows for the stability of the shoulder joint while also allowing for mobility.

## 4. Thoracic Outlet Syndrome and Shoulder Biomechanics

### 4.1. Shoulder Components and Ligaments

In those with TOS, the stability of the shoulder joint may be negatively affected by lax ligaments. Ligamentous laxity, also known as hypermobility, allows for increased mobility in joints outside of the normal range-of-motion [40]. In one study, 54% of individuals who presented with hypermobility also had symptoms of TOS [40]. If laxity is introduced, the material properties of the ligament may be altered, thus influencing the structural response of the connective tissue [41]. The viscoelastic properties of shoulder ligaments may also be further altered, due to the inherent pathophysiology of TOS, specifically with regard to the healing and treatment process [41]. During healing, there may be a recurrence of symptoms associated with TOS, due to the presence of scar tissue around the brachial plexus and subclavian vessels [42,43,44]. This scar tissue, if present near or around the shoulder ligaments, may stretch during rehabilitation treatment sessions, and add to the laxity of the healthy ligamentous tissue in the joint [45]. This, in turn, may cause cartilage degeneration in the shoulder [45]. Although it is not known whether early interventions (including physical rehabilitation) reduce ligament stretching during the healing process, early mobilization in this process may increase the cellularity, collagen content, and tensile strength of ligaments [46,47].

The presence of hypermobility in a synovial joint has been associated with an increased prevalence of premature osteoarthritis [48,49,50,51]. In those with TOS, arthritis of the first costovertebral joint was found to be the underlying etiology in 11% of individuals [52]. Conversely, joint injury or trauma is a known risk factor for osteoarthritis [53]. It is known that joint pain is a common symptom in those with TOS, hypermobility, and osteoarthritis [40,53]. Much of the pain experienced by those with TOS originates from the compression of nerves of blood vessels that innervate the shoulder musculature.

### 4.2. Shoulder Musculature

When evaluating patients with TOS, healthcare professionals typically assess the mobility of the shoulder [14]. Muscle weakness and muscle tightness can cause a plethora of issues in those diagnosed with TOS. A common feature exhibited by individuals with TOS is a flexed head position, depressed and anteriorly shifted shoulder, and protracted scapula [14,22]. This abnormal shoulder position, combined with 90° of abduction or flexion (as is commonly observed with those whose occupations require reaching, especially overhead, and repeated loading), could lead to a decrease in the costoclavicular space, increased friction of the neurovascular bundle in the subpectoral bundle, and a shortening of the sternocleidomastoid [54]. The shortened sternocleidomastoid may cause the scalenes and pectoralis muscle groups to shorten, leading to improper head and neck alignment and postural dysfunction [54].

**Figure 1:**
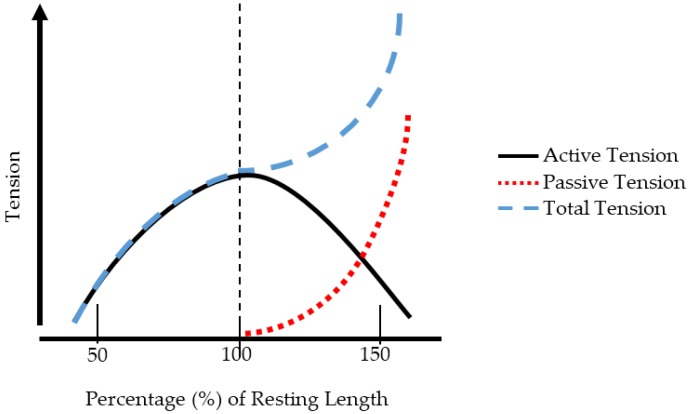
The force-length relationship characteristics for skeletal muscle.

When a muscle is chronically shortened, it cannot produce adequate force. This can be denoted by the force-length relationship (F-L), which describes the force generation characteristics for muscles. The F-L relationship is depicted in Figure 1. The active tension generated by the muscle fibers (the actin-myosin components) produces the most force at the resting length of the muscle [55]. This is a critical component of the F-L relationship. If the muscle length is too short, then the active tension will be decreased. Conversely if the muscle length is too long, there will be an insufficient number of crossbridges between actin and myosin, and force generation will be decreased [55]. The passive tension is due to the elastic components of the muscle (i.e., tendon, fascia, titin). Total tension is the combination of passive and active tension [55]. When a muscle is chronically shortened, the F-L curve shifts leftward [56]. If the muscle remains in this shortened state, the number of sarcomeres will decrease to maintain an optimal overlap between myosin and actin [56,57]. If a muscle-tendon unit is compromised, due to trauma or injury, the viscoelastic behavior of the tendon is altered. Specifically, an increase in the stiffness of the tendon is observed, resulting in a greater Young’s modulus [56]. This increase in stiffness may cause the velocity of muscle shortening to decrease during both concentric and eccentric muscle contractions, thereby reducing overall force generation capabilities. This would likely affect exercise performance during aerobic, resistance, and flexibility training. Therefore, a knowledge of general muscle mechanics, and the function of the musculature surrounding the shoulder, as well as the musculature required for proper posture, is essential. Once these concepts are understood, proper treatment plans can be prescribed for those with TOS.

## 5. Treatment Options

If an individual is diagnosed with arterial or venous TOS, surgery is typically the only option due to the severity of the injury [58]. For other forms of TOS, surgery is typically not the first option for treatment, due to the conflicting evidence regarding procedures and recovery outcomes [3]. Even if surgery is successful in the treatment of TOS, self-reported scores on a functional and pain scale are worse when compared to a normal population [12]. Nonsteroidal anti-inflammatory medication can be used to help mitigate excessive inflammatory responses, particularly early in the diagnosis. However, prolonged use of nonsteroidal anti-inflammatory medication should be avoided, due to potential health risks [59]. Muscle relaxers may be used, but due to potential addiction, they are not recommended [59]. Exercise may, therefore, be a more appropriate immediate treatment for individuals diagnosed with nTOS and non-symptomatic TOS.

### Exercise

Exercise has shown to be a useful approach in 50 to 90% of all TOS cases [6]. Although there is much variation within and between individuals, symptoms of TOS generally improve with exercise and other physical therapy techniques (e.g., manual therapy or manual adjustments) [21,60,61,62,63]. In a general exercise session, emphasis on proper scapular function during upper-body movements, breathing techniques, and head and pelvis alignment during various tasks is essential for treating TOS [36,58,64]. Resistance exercises can be performed with either resistance bands or dumbbells, and with a goal of achieving muscular endurance (i.e., low weight and high number of repetitions). Shortly after diagnosis, women and men should use 2 kg and 3 kg, respectively, if using weights [64]. However, strengthening exercises alone will not alter the pathophysiology of TOS; a combination of strengthening, stretching, and postural adjustments must all be incorporated for improvements to be observed [22,64].

Our recommendation is that exercises should initially incorporate shoulder movements ranging from 0 to 30° flexion, while maintain approximately 40° horizontal abduction. Individuals should eventually progress to shoulder movements that incorporate 45° to 90° flexion and functional overhead tasks. It is important to initially target scapular muscles (e.g., middle and lower trapezius and rhomboids) in an effort to stabilize the shoulder [64]. As patients progress, the strengthening of the serratus anterior musculature is important, but horizontal adduction should be minimized to prevent further injury [64]. Proper technique needs to be maintained throughout the rehabilitation process, as improper or inappropriate movements of other joints (e.g., excessive elbow flexion) may alter the recruitment patterns of the shoulder muscles [33]. The stretching of the scalenes and pectoralis muscles, while strengthening the muscles of cervical spine (i.e., cervical erectors, rhomboid major and minor, and lower trapezius), should be an area of focus for practitioners [54].

**Figure 2:**
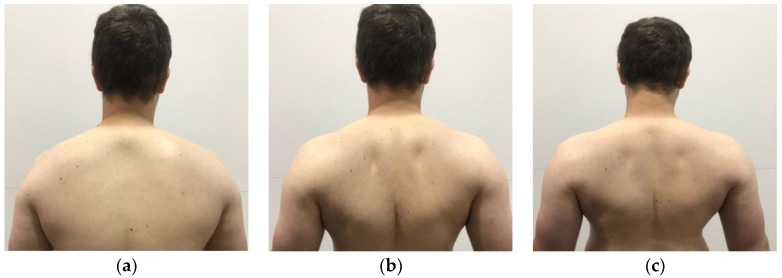
Demonstration of scapular retraction and depression in the (**a**) start position; (**b**) end position of scapular retraction; (**c**) end position of scapular depression. For scapular retraction, emphasis is placed on “pulling” the shoulder blades backwards. For scapular depression, emphasis is placed on “pulling” the shoulders back and down.

**Figure 3:**
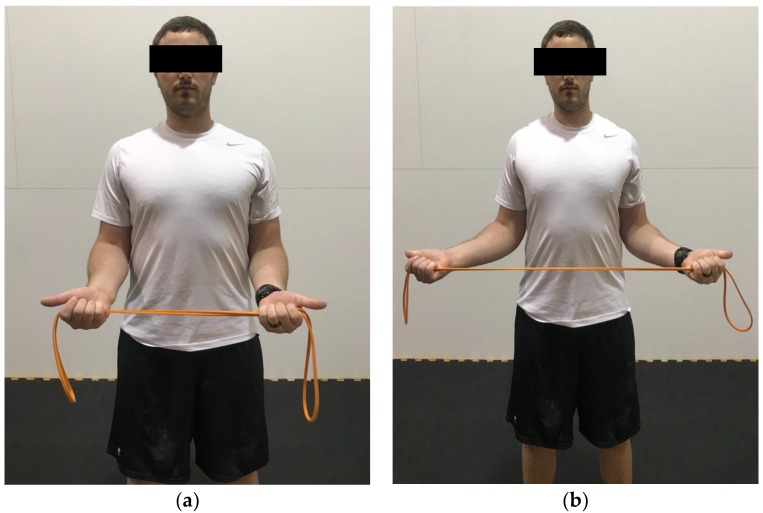
Demonstration of standing external rotation in the (**a**) start position; (**b**) end position. Hands are pronated and elbows are flexed to approximately 90°. Pull the band apart, while focusing on retracting the scapula.

**Figure 4:**
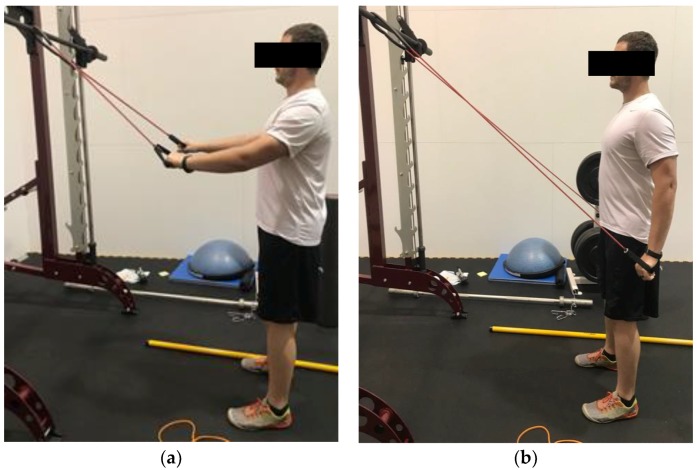
Demonstration of banded straight arm extension in the (**a**) start position; (**b**) end position. Arms start either elevated or parallel with the ground. The elbows stay slightly flexed, and the hands are brought down to the thigh while keeping the arms straight.

**Figure 5:**
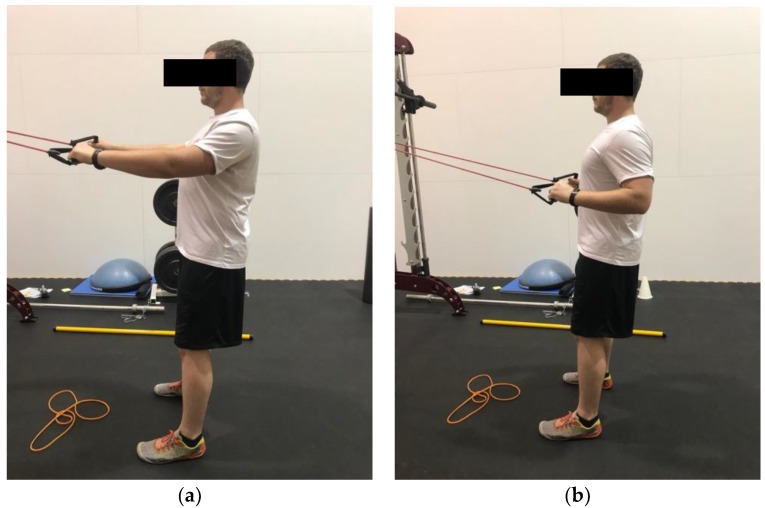
Demonstration of banded high pull in the (**a**) start position; (**b**) end position. The scapula is required first to be retracted and depressed. The band is then pulled to the chest.

**Figure 6:**
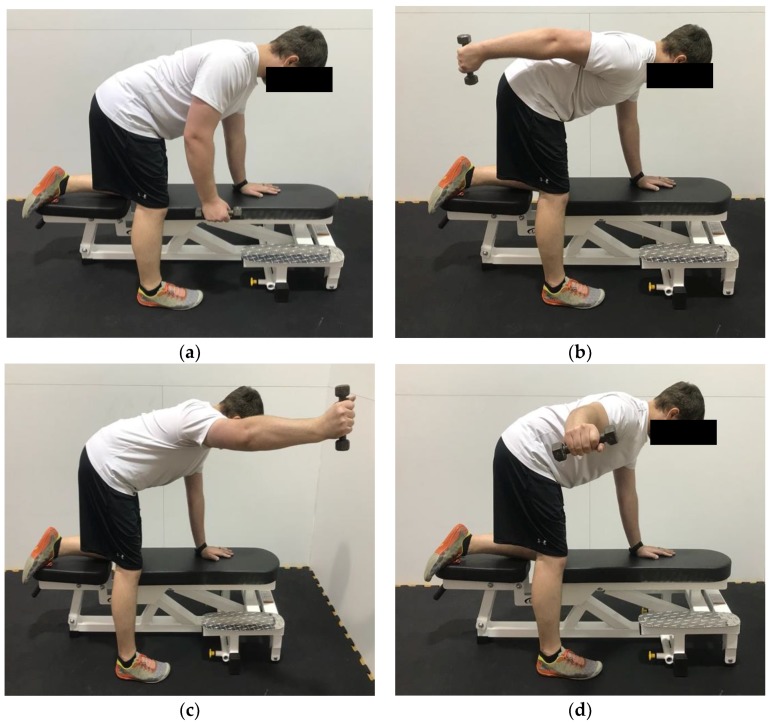
Demonstration of prone shoulder extension, abduction, and horizontal abduction in the (**a**) start position; (**b**) end position for extension; (**c**) end position for abduction; (**d**) end position for horizontal abduction. The goal in performing these exercises is to keep the scapula flush against the rib cage while moving through the various shoulder motions. This shows a modified version, utilizing a bench if a table is not available.

**Figure 7:**
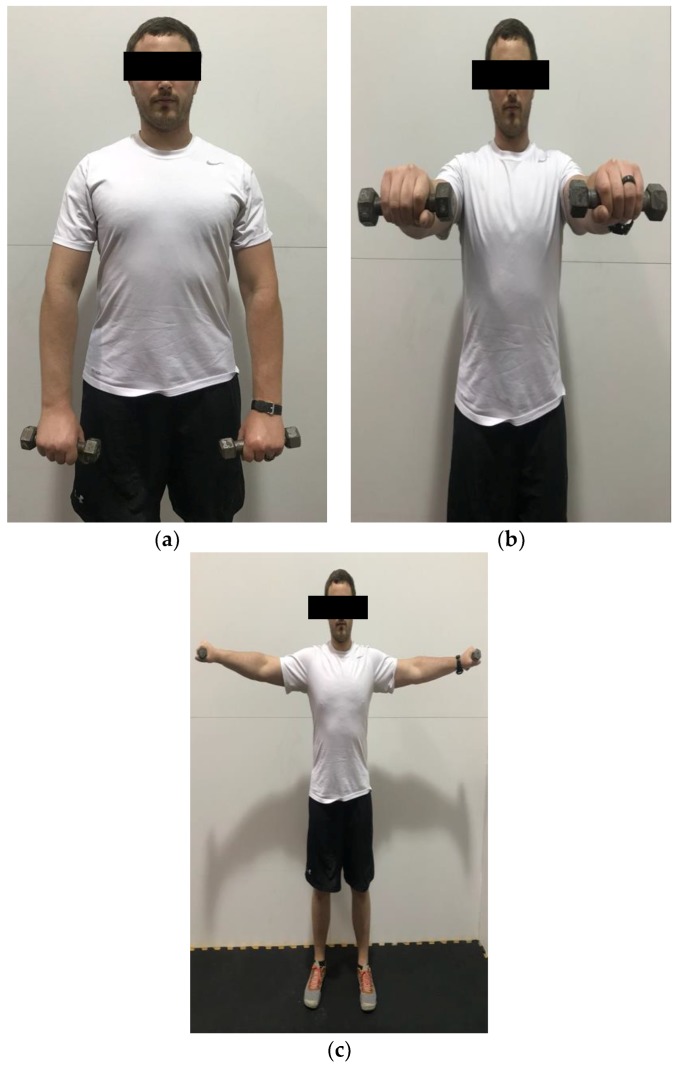
Demonstration of frontal raise and lateral raise in the (**a**) start position; (**b**) end position for frontal raise; (**c**) end position for lateral raise. Brace the abdominal muscles and slowly raise and lower the weight.

**Figure 8:**
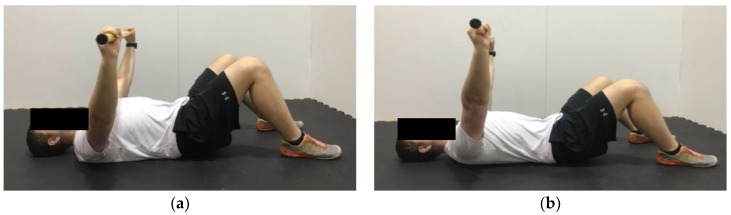
Demonstration of serratus push in the (**a**) start position; (**b**) end position. Hold the bar further than shoulder width. The goal is to avoid excessive horizontal adduction, while keeping the arms straight and pushing the bar upwards.

**Figure 9:**
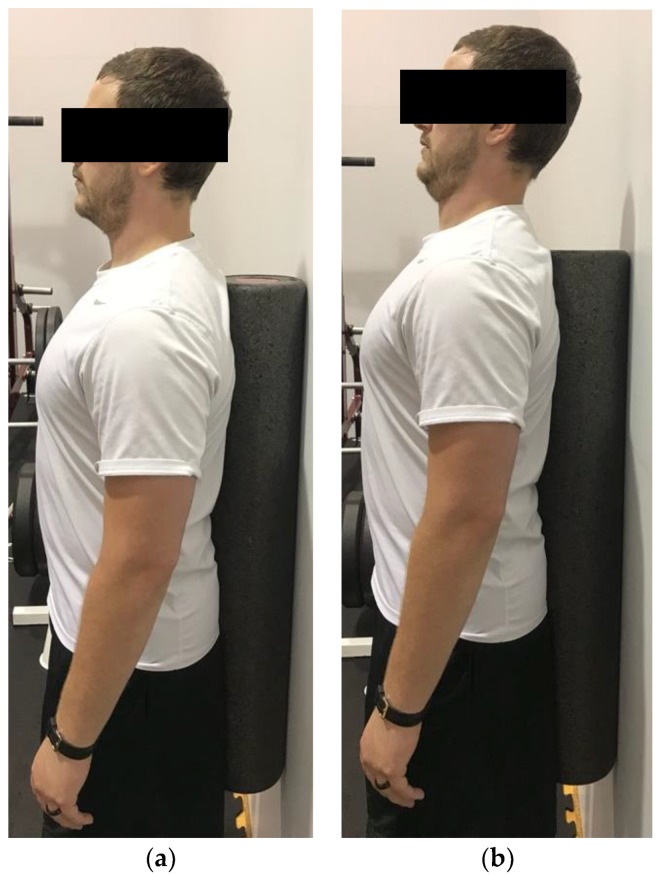
Demonstration of chin tuck in the (**a**) start position; (**b**) end position. The goal is to tuck the chin and “push” the chin into the body.

A summary of exercises targeting the shoulder muscles are displayed in Table 2, while a visual guide for these exercises are given in Figure 2, Figure 3, Figure 4, Figure 5, Figure 6, Figure 7, Figure 8 and Figure 9. These exercises may have a variety of different modifications and progressions, and it is up to the practitioner’s discretion to provide the correct exercise prescription. For example, a six-month long physical therapy program consisting of at-home exercises, stretching, postural corrections, and muscle recruitment patterns, primarily focusing on the neck and shoulder, can alleviate symptoms associated with TOS [65].

## 6. Conclusions

The complexity of TOS is mirrored by the complexity of the shoulder. Improper shoulder positioning and muscle weakness can have a larger effect on other tissues in the body. Exercise is a conservative and effective approach with regard to the treatment of TOS. More research is needed to determine the exact etiology of various forms of TOS so treatments, such as exercise, may be more effectively utilized.

## Figures and Tables

**Table 1:** Summary of other significant shoulder musculature.

Muscle	Origin	Insertion	Function
Latissimus dorsi [33]	- Thoracic vertebrae - Sacral vertebrae - Posterior iliac crest	Intertubercular groove of humerus	Shoulder adduction and internal rotation
Deltoid [26,32,33]	- Clavicle - Acromion - Spine of scapula	Deltoid tuberosity	- Anterior head: flexion of humerus - Medial head: Abduct shoulder and maintain humerus into the glenohumerual joint - Posterior head: extension of the humerus
Trapezius [32,35]	- Base of occipital bone-Ligamentum nuchae-C7–T12 vertebrae	- Acromion - Spine of scapula - Deltoid tubercle - Clavicle	Scapular elevation, depression, and retraction
Serratus anterior [32]	Ribs 1–8	Medial border of the scapula	- Protraction of scapula-Prevents scapular winging and tilt
Major/minor rhomboids [32]	- C6–C7 vertebrae (minor) - T1–T4 (major) - Supraspinous ligament	Medial border of the scapula	Adduction of the scapula
Major/minor pectoralis [32]	- Sternum and clavicle (major) - Ribs 3–5 (minor)	- Intertubercular groove (major) - Greater tubercle of the humerus (major) - Coracoid process of the scapula (minor)	- Adduction and medial rotation of shoulder (major) - Adduction and protraction of scapula (minor)
Long head of biceps brachii [23,28,30]	Supraglenoid tubercle of scapula	Intertubercular groove	Stabilization from anterior-posterior translations

**Table 2:** Summary of suggested exercises to target shoulder and scapular musculature.

Exercise	Muscles Targeted	Exercise	Muscles Targeted
Scapular retraction	- Rhomboids - Trapezius	Prone shoulder extension, abduction, horizontal abduction “I’s, Y’s and T’s”	- Rhomboids - Trapezius - Supraspinatus - Infraspinatus - Deltoid - Latissimus dorsi - Teres major
Scapular depression	- Trapezius (lower) - Latissimus dorsi - Rhomboids	Frontal raise	Deltoid
Standing external rotation “No Money”	- Trapezius (lower) - Infraspinatus - Teres minor - Subscapularis	Lateral raise	- Deltoid - Supraspinatus - Trapezius
Straight arm extension	- Latissimus dorsi - Teres major - Triceps brachii	Serratus push	Serratus anterior
Banded high rows	- Latissimus dorsi - Trapezius - Rhomboids - Teres major/minor	Chin tuck	Trapezius
